# Breaking the cellular defense: the role of autophagy evasion in *Francisella* virulence

**DOI:** 10.3389/fcimb.2024.1523597

**Published:** 2024-12-24

**Authors:** Pavla Pavlik, Eva Velecka, Petra Spidlova

**Affiliations:** ^1^ Department of Molecular Pathology and Biology, Military Faculty of Medicine, University of Defence, Hradec Kralove, Czechia; ^2^ Institute of Organic Chemistry and Biochemistry, Czech Academy of Sciences, Prague, Czechia

**Keywords:** *Francisella*, autophagy, virulence, bacterial pathogenesis, host-pathogen interaction

## Abstract

Many pathogens have evolved sophisticated strategies to evade autophagy, a crucial cellular defense mechanism that typically targets and degrades invading microorganisms. By subverting or inhibiting autophagy, these pathogens can create a more favorable environment for their replication and survival within the host. For instance, some bacteria secrete factors that block autophagosome formation, while others might escape from autophagosomes before degradation. These evasion tactics are critical for the pathogens’ ability to establish and maintain infections. Understanding the mechanisms by which pathogens avoid autophagy is crucial for developing new therapeutic strategies, as enhancing autophagy could bolster the host’s immune response and aid in the elimination of pathogenic bacteria. *Francisella tularensis* can manipulate host cell pathways to prevent its detection and destruction by autophagy, thereby enhancing its virulence. Given the potential for *F. tularensis* to be used as a bioterrorism agent due to its high infectivity and ability to cause severe disease, research into how this pathogen evades autophagy is of critical importance. By unraveling these mechanisms, new therapeutic approaches could be developed to enhance autophagic responses and strengthen host defense against this and other similarly evasive pathogens.

## Graphical Abstract

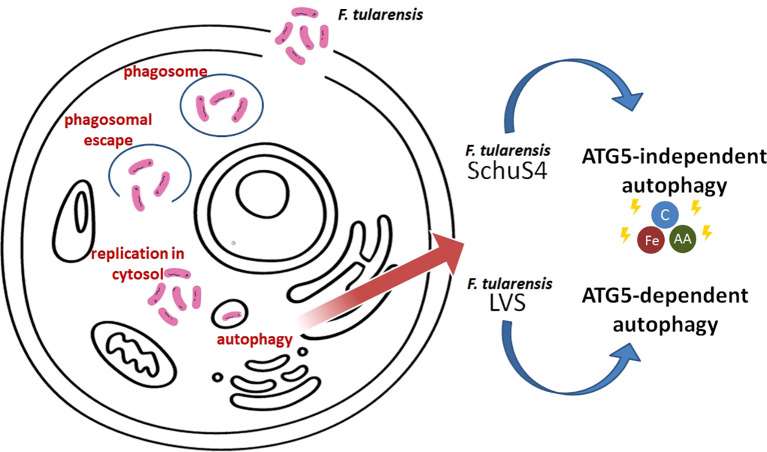

## Introduction

*Francisella* is a genus of gram-negative bacteria that includes the highly virulent species *F. tularensis*, known for causing tularemia in humans and other mammals. Researches have revealed that *F. tularensis* can evade the host’s immune response by escaping the phagosome. Instead of being targeted for degradation, the bacterium can replicate within the host cell’s cytoplasm, where it is protected from immune responses. One intriguing aspect of the interaction between *F. tularensis* and its host cells is its ability to dampen the host’s cellular processes, including autophagy. The bacterium has developed mechanisms to manipulate the host cell’s autophagy machinery promoting its intracellular survival. Studies have shown that *F. tularensis* can inhibit the fusion of autophagosomes with lysosomes, where the contents are typically degraded. By preventing this fusion, the bacterium can avoid destruction and create a favorable intracellular niche for replication. Understanding the interplay between *F. tularensis* and autophagy is crucial for developing more effective treatments and vaccines against tularemia.

### The basics of autophagy

Autophagy is a fundamental cellular process responsible for the degradation and recycling of cellular components. This mechanism is crucial for maintaining cellular homeostasis, responding to stress conditions, and survival during nutrient deprivation. Autophagy involves the formation of autophagosomes, double-membraned vesicles that engulf damaged organelles, misfolded proteins, and pathogens for degradation in the lysosomes (Levine and Kroemer, 2019).

The process is highly regulated and can be triggered by various cellular signals, including nutrient levels, growth factors, and intracellular energy status. The regulation of autophagy is mediated by a complex network of signaling pathways, with the mammalian target of rapamycin (mTOR) pathway playing a central role in inhibiting autophagy under nutrient-rich conditions. Conversely, activation of AMP-activated protein kinase (AMPK) promotes autophagy by inhibiting mTOR under nutrient scarcity (Kim et al., 2011).

Autophagy not only helps in the removal of cellular debris but also plays a crucial role in defense mechanisms against infections by degrading intracellular pathogens through a process known as xenophagy (Deretic et al., 2013). Nevertheless, some pathogens together with *F. tularensis* can affect host cellular processes, including autophagy, to their advantage.

### Mechanisms and pathways

Autophagy is a cellular degradation process crucial for maintaining cellular homeostasis by removing damaged organelles and misfolded proteins. There are three primary types of autophagy: macroautophagy, microautophagy, and chaperone-mediated autophagy (CMA), each with distinct mechanisms and functions.

**Macroautophagy** is the most extensively studied form of autophagy and involves the formation of double-membrane vesicles called autophagosomes. These vesicles engulf cytoplasmic material and then fuse with lysosomes, where the content is degraded and recycled. The process begins with the nucleation and expansion of the isolation membrane, which requires the expression of various autophagy-related proteins (ATGs). Key regulators of this pathway include the ULK1 complex, Beclin-1, and the ATG8/LC3 family proteins. This type of autophagy is highly regulated by nutrient availability and cellular stress, playing a critical role in cell survival during starvation and other stress conditions (Yamamoto and Matsui, 2024).

One of the primary roles of macroautophagy in host defense is the direct elimination of intracellular pathogens. This process, termed xenophagy, targets bacteria, viruses, and other pathogens for degradation. Xenophagy, also known as selective autophagy of pathogens, is a host defense mechanism where intracellular pathogens are recognized, sequestered within double-membraned autophagosomes, and subsequently degraded after fusion with lysosomes. This process is essential for maintaining cellular homeostasis and protecting the host from infections by targeting and eliminating invasive microbes such as bacteria, viruses, and parasites (Yuk et al., 2012). Xenophagy not only removes these pathogens but also facilitates antigen presentation, enhancing the adaptive immune response. The process of xenophagy begins with the recognition of intracellular pathogens through pattern recognition receptors (PRRs) that identify pathogen-associated molecular patterns (PAMPs). By delivering pathogen-derived antigens to major histocompatibility complex (MHC) molecules, autophagy enhances the presentation of these antigens to T cells. This process is critical for the immune system to recognize and respond to infections effectively (Van Kaer et al., 2019). Autophagy contributes to the presentation of viral antigens on MHC class II molecules, thus aiding in the activation of CD4+ T cells during viral infections (Schmid et al., 2007). Once recognized, adaptor proteins such as p62/SQSTM1 link the pathogen to autophagic machinery by binding to ubiquitinated microbial proteins and recruiting autophagy-related proteins like LC3 (Zheng et al., 2009). This recruitment ensures the encapsulation of the pathogen within autophagosomes. However, many pathogens have evolved sophisticated strategies to evade or manipulate xenophagy. Some escape recognition by autophagic receptors, while others inhibit autophagosome formation or prevent their fusion with lysosomes (Mao and Klionsky, 2016).

In addition to direct targeting pathogens, autophagy plays a significant role in modulating inflammatory responses. By degrading damaged organelles and excess proteins, autophagy prevents the accumulation of cellular debris that can trigger inflammation. Furthermore, autophagy regulates the production of pro-inflammatory cytokines, such as IL-1β, by controlling the activation of the inflammasome (Harris et al., 2011; Claude-Taupin et al., 2018; Iula et al., 2018).

**Microautophagy** involves the direct engulfment of cytoplasmic material by the lysosome itself through invagination, protrusion, or septation of the lysosomal membrane. Unlike macroautophagy, this process does not require the formation of autophagosomes. Instead, the lysosomal membrane engulfs small portions of the cytoplasm, including organelles and proteins, which are then degraded within the lysosome. Microautophagy is less well understood than macroautophagy but is believed to be crucial for maintaining organelle size and number, as well as for responding to nutrient depletion (Yamamoto and Matsui, 2024).

**Chaperone-mediated autophagy (CMA)** is distinct from both macroautophagy and microautophagy since it specifically targets soluble cytosolic proteins for degradation. This selectivity is mediated by chaperone proteins, particularly heat shock protein 70 (Hsp70), which recognize and bind to specific KFERQ-like motifs in the substrate proteins. These substrates are then translocated across the lysosomal membrane via the lysosomal-associated membrane protein 2A (LAMP-2A). CMA is involved in the degradation of long-lived proteins and plays a significant role in cellular quality control and stress response (Orenstein and Cuervo, 2010; Kaushik and Cuervo, 2018).

### Pathogenic bacteria and their influence on autophagy

Some pathogenic bacteria inhibit autophagy to avoid degradation. They employ various strategies to overcome autophagy, such as inhibiting autophagosome formation, evading recognition, and preventing acidification. This section summarizes several bacterial pathogens and their effectors (see **Table 1**).

**Table 1:** Bacteria and their effector proteins affecting autophagy.

Bacteria	Effector proteins	Positive/negative modulation of the autophagy	References
*Salmonella*	SopB	Negative	(Tattoli et al., 2012; Chatterjee et al., 2023)
	SsrB, SsaV, SseG, SseF, SseL, SpvB	Negative	(Mesquita et al., 2012; Ganesan et al., 2017; Feng et al., 2018)
	CdtB, Type II L-asparaginase, SipD, β-OMP	Positive	(Hernandez et al., 2003; Williams et al., 2015; Torres et al., 2016; Chaudhary et al., 2018)
*Shigella*	IcsB	Negative	(Baxt and Goldberg, 2014; Ashida et al., 2015)
*Legionella*	RavZ, LpSp1, Lpg1137, Lpg2936, SidE, SdeA, SdeB, SdeC	Negative	(Degtyar et al., 2009; Choy et al., 2012; Bhogaraju et al., 2016; Rolando et al., 2016; Arasaki et al., 2017; De Leon et al., 2017; Pinotsis and Waksman, 2017; Abd El Maksoud et al., 2019; Omotade and Roy, 2020)
	LegA9, SetA	Positive	(Khweek et al., 2013; Beck et al., 2020)
*Brucella*	BtpB, NyxA, NyxB	Negative	(Li et al., 2022; Louche et al., 2023)
*Mycobacterium*	PknG, SapM, PE_PGRS20, PE_PGRS47, PtpA, ESAT-6, CFP-10	Positive/Negative	(Tan et al., 2006; Wong et al., 2011; Kim et al., 2020; Strong et al., 2021; Ge et al., 2022; Zhang et al., 2024)
*Listeria*	PlcA, PlcB, ActA, LLO, InlK	Negative	(Smith et al., 1995; Schnupf and Portnoy, 2007; Yoshikawa et al., 2009a, Yoshikawa et al., 2009b; Dortet et al., 2011, Dortet et al., 2012; Mitchell et al., 2015)
*Streptococcus*	SpyCEP, SpeB,	Negative	(Barnett et al., 2013; Bergmann et al., 2022)
*Yersinia*	YopB, YopD, YopJ (YopP), YopM, YopE, YopT, YopH, YpkA (YopO), YopK (YopQ)	Negative	(Seabaugh and Anderson, 2024)
	Unknown plasmid-borne effectors	Positive	(Lemarignier and Pizarro-Cerdá, 2020)
*Pseudomonas*	ExoS	Negative	(Rao et al., 2021)
*Bacillus*	Edtx	Negative	(Shahnazari et al., 2011)
*Vibrio*	Ctx,	Negative	(Huang and Brumell, 2014)
	VCC,	Positive/Negative	(Gutierrez et al., 2007)
	MakA	Positive/Negative	(Corkery et al., 2021; Jia et al., 2022)
*Enterococcus*	LTA	Positive	(Lin et al., 2018)
*Francisella*	IglC	Negative	(Santic et al., 2005)
	PdpC, PdpD	Negative	(Eshraghi et al., 2016)
	OpiA	Negative	(Ledvina et al., 2018)

For instance, ***Salmonella enterica* serovar Typhimurium**, a common cause of foodborne illness, secretes effector proteins through its type III secretion system (T3SS) to inhibit autophagy. The bacterial effector protein SopB disrupts the formation of autophagosomes by interfering with the host cell’s signaling pathways (Tattoli et al., 2012; Chatterjee et al., 2023). This inhibition allows *Salmonella* to reside within a modified vacuole, where it can replicate and evade host immune responses. Other studies have also confirmed that effectors of SPI-2 (*Salmonella* pathogenicity island 2) T3SS as well as *Salmonella* virulence plasmids are important for bacterium to escape phagocytosis (Wu et al., 2020). For example, SsrB and SsaV effector proteins are responsible for activating mTOR through disruption of AMPK signaling (Ganesan et al., 2017). Other effector proteins, SseG and SseF, block Rab1 activity in host cell, which results in reduction of autophagosome formation and effective bacterial replication in cytoplasm (Feng et al., 2018). SseL acts as a deubiquitinase, which removes ubiquitin markers from *Salmonella*-infected cells, thus enabling bacterial replication instead of autophagic degradation (Mesquita et al., 2012). On the other hand, there exist *Salmonella´s* proteins that are involved in autophagy induction. L-asparaginase hydrolyzes the L-asparagine, thus T cells are not activated, which results in mTOR signaling inhibition and autophagy induction (Torres et al., 2016). Among other autophagy inducing proteins belong β-barrel outer membrane protein (β-OMP), *Salmonella* invasive protein D (SipD) and cytoskelethal distending toxin B (CdtB) (Hernandez et al., 2003; Williams et al., 2015; Chaudhary et al., 2018).

***Shigella***, a genus of bacteria responsible for causing dysentery, has developed sophisticated mechanisms to evade and manipulate the host’s immune responses, particularly autophagy (Phalipon and Sansonetti, 2007). *Shigella* can inhibit the formation of autophagosomes, the vesicles responsible for sequestering pathogens. This inhibition prevents the bacteria from being engulfed and degraded. *Shigella* achieves this through the secretion of effector proteins via its T3SS, which interfere with autophagy signaling pathways. Even if *Shigella* is initially captured by autophagosomes, it can escape before being degraded. The effector protein IcsB, secreted by *Shigella*, helps it evade autophagic recognition. IcsB interferes with the recruitment of autophagy-related proteins, such as ATG5, which are necessary for the formation of autophagosomes (Baxt and Goldberg, 2014; Ashida et al., 2015).

***Legionella pneumophila***, the causative agent of Legionnaires’ disease, hijacks the autophagic machinery through the irreversibly inactivating ATG8 proteins to create a replicative niche known as the *Legionella*-containing vacuole (LCV). *Legionella* secretes many effector proteins (Thomas et al., 2020), such as RavZ, that modulate autophagy to divert autophagic vesicles away from the lysosomal degradation pathway, thereby creating a favorable environment for bacterial replication (Choy et al., 2012).

LpSpl, a *Legionella* effector, mimics the enzymatic activity of sphingosine-1-phosphate lyase 1 (SGPL1), a key regulator of sphingolipid metabolism. LpSpl’s localization to mitochondria and the endoplasmic reticulum suggests a complex interplay with host metabolic pathways (Degtyar et al., 2009; Rolando et al., 2016). By reducing sphingosine levels, which are known to stimulate autophagy (Dall’Armi et al., 2013), LpSpl inhibits autophagosome biogenesis. This inhibition is dependent on its enzymatic activity, as mutants lacking functional active sites do not affect autophagy (Rolando et al., 2016). Despite its ability to modulate autophagy, LpSpl does not significantly impact bacterial replication in macrophages or amoebae but is required for optimal replication in mouse models (Rolando et al., 2016).

Lpg1137, a serine protease, disrupts autophagosome biogenesis by degrading Syntaxin 17 (Stx17), a key SNARE protein (soluble N-ethylmaleimide-sensitive factor attachment protein receptors) involved in autophagosome-lysosome fusion and autophagy initiation (Hamasaki et al., 2013; Kumar et al., 2019). By cleaving Stx17, Lpg1137 prevents the formation of LC3- and ATG14-positive puncta, essential for autophagosome formation (Arasaki et al., 2017). Interestingly, bioinformatic analysis suggests Lpg1137 resembles mitochondrial SLC25 carrier proteins, though its exact mechanism requires further structural characterization (Gradowski and Pawłowski, 2017).

The effector Lpg2936 modulates host autophagy by inducing epigenetic changes in the promoter regions of key autophagy genes, such as ATG7 and LC3B. By methylating the GATC motif in these promoters, Lpg2936 suppresses autophagosome formation, indirectly promoting bacterial replication (Abd El Maksoud et al., 2019). While this highlights its role as a transcriptional regulator, its potential autophagy-independent roles remain under investigation (Pinotsis and Waksman, 2017).

In contrast to other effectors, LegA9 enhances the recruitment of SQSTM-1 to *Legionella*-containing vacuoles (LCVs), promoting their recognition for autophagic clearance (Khweek et al., 2013). However, this effector does not directly activate autophagy, and its role appears to be more relevant in alternative hosts, suggesting an evolutionary trade-off in mammalian infections (Price et al., 2020).

The SidE family of effectors (SidE, SdeA, SdeB, and SdeC) inhibits the recruitment of autophagy adapters such as SQSTM-1 to LCVs by generating unique ubiquitin linkages (Bhogaraju et al., 2016; Omotade and Roy, 2020). Furthermore, these effectors promote TFEB (transcription factor EB) nuclear translocation, which upregulates autophagy and lysosomal genes, suggesting a dual role in balancing nutrient acquisition and autophagy inhibition (De Leon et al., 2017). Temporal regulation appears essential, as SidE activity is blocked during co-expression with other *Legionella* effectors (De Leon et al., 2017).

SetA glucosylates TFEB, preventing its cytoplasmic retention and promoting its nuclear translocation, thereby inducing autophagic gene expression (Beck et al., 2020).

***Brucella abortus***, a pathogen responsible for brucellosis, resides in a membrane-bound compartment called the *Brucella*-containing vacuole (BCV) upon entry into host cells (Celli, 2019). *Brucella* inhibits the fusion of intermediate BCVs with late endosomes and lysosomes, which are critical steps in the autophagic process. Despite preventing full fusion with lysosomes, BCVs acquire several markers of late endosomes, including Rab7, a small GTPase, and its effector Rab-interacting lysosomal protein (RILP). This interaction allows *Brucella* to manipulate the endocytic pathway while avoiding degradation (Starr et al., 2008). *Brucella* effector like BtpB interferes with the host’s autophagic signaling pathways, allowing the bacteria to persist in a replication-permissive environment (Li et al., 2022). Interestingly, some bacteria can both inhibit and exploit autophagy at different stages of infection. The *Brucella* effector proteins NyxA and NyxB modulate host autophagy by targeting the SUMO-specific protease SENP3, which regulates autophagy-related protein de-SUMOylation. By disrupting SENP3 activity, NyxA and NyxB inhibit xenophagy, allowing *Brucella* to evade autophagic degradation and establish a replicative niche. This interference also dampens inflammatory responses associated with autophagy, promoting bacterial survival and persistence within host cells (Louche et al., 2023).

***Mycobacterium tuberculosis***, the bacterium responsible for tuberculosis, initially inhibits autophagy to prevent its destruction within macrophages. However, during later stages of infection, *M. tuberculosis* can exploit the autophagic process to access nutrients and enhance its survival. This dual manipulation underscores the complexity of bacterial interactions with the autophagic machinery (Castillo et al., 2012). *Mycobacterium* can activate the mTOR pathway, a key negative regulator of autophagy. By maintaining mTOR activity, *Mycobacterium* prevents the initiation of autophagy, hindering the formation of autophagosomes (Singh and Subbian, 2018). This is crucial because the initiation of autophagy is dependent on the inhibition of mTOR, which normally suppresses the activity of the ULK1 complex, essential for the nucleation of autophagosomes (Kim et al., 2011). Bacterium secretes proteins such as PknG, a serine/threonine kinase, which inhibits the maturation of phagosomes. PknG prevents the acidification and fusion of Mtb-containing phagosomes with lysosomes, thereby blocking their transformation into autophagolysosomes where the bacteria would be degraded (Ge et al., 2022). *Mycobacterium* also secretes SapM, which interacts with the adaptor protein Raptor that is involved in the mTOR pathway. SapM causes the dephosphorylation of Raptor and this interaction results in mTORC1 hyperactivity, which in turn inhibits autophagy (Zhang et al., 2024). Mycobacterial proteins PE_PGRS20 and PE_PGRS47 have been shown to interact with host autophagy proteins. These interactions can modulate autophagic flux, ensuring that the autophagy process is altered in a way that favors bacterial survival rather than its degradation. These proteins interact directly with Ras-related protein Rab1A - a multifunctional regulator in the autophagy pathway (Strong et al., 2021). Phagosomal maturation through fusion with lysosomes relies, besides others, on vacuolar ATPase, which acidifies the phagosomal lumen by hydrolyzing ATP. *Mycobacterium* inhibits host vacuolar ATPase using mechanisms involving the mycobacterial secreted phosphatase PtpA that interacts with vacuolar ATPase to enhance bacterial survival and pathogenicity (Wong et al., 2011; Kim et al., 2020). Two mycobacterial proteins ESAT-6 and CFP-10 are also secreted and plays a critical role in preventing phagolysosomal fusion, thereby aiding in the intracellular survival of *Mycobacterium* (Tan et al., 2006). Interaction of *Mycobacterium* with host autophagy is very well described in (Kim et al., 2020).

***Listeria monocytogenes***, a facultative intracellular pathogen, avoids autophagy by expressing two key determinants of pathogenesis: secreted phosphatidylinositol-specific phospholipases C (PlcA) (Mitchell et al., 2015), broad-range phospholipase C (PlcB) (Smith et al., 1995), a surface protein (ActA) (Yoshikawa et al., 2009a, Yoshikawa et al., 2009b) and pore-forming cytolysin listeriolysin O (LLO) (Schnupf and Portnoy, 2007). These factors allow the bacterium to escape from phagosomes, grow in the host cytosol, and evade the autophagic response (Mitchell et al., 2015, 2018). In addition to these proteins, the surface-associated protein InlK, encoded by the *lmo1290* gene, has been identified to play a crucial role in *Listeria’s* ability to evade autophagy (Dortet et al., 2011). A yeast two-hybrid assay revealed that major vault protein (MVP), a highly abundant component of the eukaryotic cytoplasm, is a potential interacting partner of InlK (Dortet et al., 2011). InlK recruits MVP to coat the surface of *Listeria* so that the bacterium can escape autophagic recognition (Dortet et al., 2012).

In the case of ***Streptococcus***, SpyCEP (Streptococcal pyrogenic exotoxin B cleaving enzyme) and SpeB (Streptococcal cysteine protease B) are critical factors to evade the host’s autophagic defenses, facilitating their survival and proliferation within host cells. SpyCEP is an interleukin-8 protease that is highly upregulated during invasive streptococcal infections. This protease cleaves and inactivates IL-8, a key chemokine involved in recruiting immune cells to the site of infection. By doing so, SpyCEP helps bacteria evade the immune response, including autophagy, which is a critical host defense mechanism (Bergmann et al., 2022). SpeB is a cysteine protease that is involved in the degradation of host cell proteins and immune modulators. SpeB degrades p62, NDP52, and NBR1. These proteins are essential components of the autophagy machinery, functioning as adaptors that help in recognizing and targeting bacteria for autophagic degradation. By degrading these adaptors, SpeB effectively inhibits the autophagic process within the host cell cytosol (Barnett et al., 2013).

The uptake of ***Yersinia*** by host cells triggers autophagy-related processes, but the specific pathways and outcomes vary depending on the cell type and *Yersinia* species. For example, *Y. pseudotuberculosis* has been shown to induce classical autophagy in macrophages and a variant called LC3-assisted phagocytosis in epithelial cells (Moreau et al., 2010). However, these autophagic processes do not effectively eliminate the bacteria; instead, they may support bacterial survival within host cells. The study of (Ligeon et al., 2014) found that a subset of intracellular *Y. enterocolitica* localizes to autophagosomal compartments within epithelial cells. Interestingly, the autophagy triggered by *Y. enterocolitica* did not eliminate the bacteria but rather supported their intracellular survival and multiplication. This process differed from LC3-assisted phagocytosis and resembled classical autophagy, involving core components of the autophagic machinery. The increased intracellular replication of *Y. enterocolitica* due to autophagy was also associated with enhanced extracellular release of the bacteria. These findings suggest that *Y. enterocolitica* may exploit the canonical macroautophagy pathway to promote its intracellular replication and eventual escape from infected epithelial cells (Ligeon et al., 2014; Valencia Lopez et al., 2019). *Y. pestis* has been shown to reside in phagosomes that acquire certain markers of late endosomes or lysosomes but do not undergo the typical acidification process. It was demonstrated that within naive macrophages, the vacuoles containing *Yersinia* fail to acidify. This lack of acidification is crucial for the bacteria’s survival, as it prevents the activation of lysosomal enzymes that would otherwise degrade the pathogen (Pujol et al., 2009). *Yersinia* produces a variety of effector proteins that play critical roles in the bacterium’s pathogenicity, particularly by disrupting host cell responses. These proteins—YopB, YopD, YopJ (known as YopP in *Y. enterocolitica*), YopM, YopE, YopT, YopH, YpkA (referred to as YopO in *Y. enterocolitica*), and YopK (YopQ in *Y. enterocolitica*)—interfere with the host’s immune defenses. By targeting and inhibiting key cellular processes, these Yop proteins help *Yersinia* survive and proliferate within the host (Seabaugh and Anderson, 2024).

It has been demonstrated that ***Pseudomonas aeruginosa*** infection also leads to the induction of autophagy (Yuan et al., 2012) but the question has arisen if *P. aeruginosa*, an extracellular pathogen, could modulate autophagy for its own benefit. The study of (Rao et al., 2021) has revealed that this pathogen affects the host defense pathway using T3SS. This secretion system could be used for the injection of up to four cytotoxins produced by *P. aeruginosa* (Hauser, 2009). The only toxin among these secreted proteins, which could dampen autophagy, is ExoS. Its mode of action is inhibition of mTOR by ADP ribosylation of Ras and concurrently inhibition of the autophagy process through repression of Vps34 kinase activity (autophagy–associated) via ADP ribosylation (Rao et al., 2021).

***Bacillus anthracis*** produces Edema toxin (Edtx), which is a cAMP-elevating and thus capable of inhibiting autophagy as well as cholera toxin (Ctx) from ***Vibrio cholerae*** (Shahnazari et al., 2011; Huang and Brumell, 2014). Apart from Ctx, *V. cholerae* produces several other toxins. The *V. cholerae* cytolysin (VCC) protein is a key virulence factor that can disrupt host cell membranes by forming transmembrane pores, leading to cell lysis or triggering various cellular stress signaling. VCC can induce an autophagic response that leads to incomplete or stalled autophagic flux. While autophagosomes are formed in response to VCC, their maturation into autolysosomes—where degradation occurs—may be impaired, resulting in an accumulation of autophagosomes without effective breakdown of their contents (Gutierrez et al., 2007). MakA (Motility-associated killing factor A) interacts with the cellular membrane, leading to pore formation and disruption of membrane integrity. MakA is taken up by host cells, leading to the formation of cholesterol-rich membrane aggregates in a pH-dependent manner in endolysosomes, which triggers a non-canonical autophagy pathway with unconventional LC3 lipidation on these membranes (Corkery et al., 2021; Jia et al., 2022).

***Enterococcus faecalis***, a gram- positive opportunistic invasive bacterium and a member of human intestinum microbiota (Klare et al., 2001) has been shown to induce formation of autophagosomes in small intestinal epithelial cells (Benjamin et al., 2013). Conversely, studies by Zou and Shankar (2014) demonstrated that *E. faecalis* infection activates PI3K/Akt signaling pathway in host cell, potentially contributing to autophagy inhibition. Further, their research revealed that following internalization, the *Enterococcus*-containing vacuole (ECV) is a single-membrane organelle that resists acidification (Zou and Shankar, 2016). In contrast, Lin et al. (2018) suggested that *E. faecalis* lipoteichoic acid (LTA) efficiently activates macrophage autophagy. This activation is achieved by p-Akt and p-mTOR inhibition and the process is dependent on Beclin1.

## Francisella tularensis

### *Francisella* species and pathogenesis

*F. tularensis*, a causative agent of a potentially lethal zoonotic disease tularemia, is a gram-negative facultative intracellular bacterium (Maurin, 2020). With an extremely low infectious dose (fewer than 10 colony-forming units, CFU), it is considered one of the most infectious pathogens described (Travis et al., 2021). Therefore, due to its high virulence, and multiple transmission routes with easy dissemination, the U.S. Centers for Disease Control and Prevention (CDC) classifies it as a Tier 1 Select Agent with the potential to be used as a biological weapon (Rowe and Huntley, 2015).

Currently, three subspecies are distinguished based on their metabolic characteristics, and virulence differences, such as *F. tularensis*, subsp. *tularensis* (type A), *F. tularensis* subsp. *holarctica* (type B), and *F. tularensis* subsp. *mediasiatica*. However, only *F. tularensis* subsp. *tularensis* and *holarctica* are known to cause tularemia in healthy individuals (Degabriel et al., 2023). While the type A strain primarily occurs on the ground in North America, the type B strain is mainly found in countries across the Northern Hemisphere (Rowe and Huntley, 2015). Although direct human-to-human transmission has not been reported, transmission through solid organ transplantation occurred in the United States in 2017, resulting in the death of one recipient (Nelson et al., 2019).

The pathogenicity of *F. tularensis* is primarily attributed to its ability to replicate and survive within various eukaryotic cells, especially macrophages. Infections of other cells, such as dendritic cells, hepatocytes, neutrophils, or endothelial cells, have also been documented (Bröms et al., 2010; Celli and Zahrt, 2013). During infection, the pathogen is engulfed by the macrophage through an asymmetric pseudopod loop, a process known as a,looping phagocytosis” (Clemens et al., 2004). Subsequently, the pathogen resides within a *Francisella*-containing vacuole (FCV), preventing phagolysosomal fusion and escaping into the nutrition-rich cytosol, where a massive replication occurs. Eventually, this process leads to cell apoptosis and infection of surrounding macrophages, thereby spreading the infection (Pechous et al., 2009; Celli and Zahrt, 2013; Ramakrishnan, 2017). The mechanisms of phagosome escape are not fully understood yet, but a gene cluster known as the *Francisella* pathogenicity island (FPI) has been identified as a key factor, encoding proteins essential for the constitution of the atypical type VI secretion system (T6SS) (Clemens et al., 2015; Rigard et al., 2016; Spidlova and Stulik, 2017). Interestingly, none of those proteins possess properties of cytolysins, pore-forming toxins, or hydrolytic enzymes, suggesting a novel bacterial escape mechanism (Bröms et al., 2010). In addition to FPI proteins, *F. tularensis* virulence is critically dependent on several other factors, including MglA, SspA, PigR (also known as FevR), ppGpp, (Lauriano et al., 2004; Wrench et al., 2013) and the HU protein (Stojkova et al., 2018, Stojkova et al., 2019; Stojkova and Spidlova, 2022). These proteins, along with various other transcription factors (Spidlova et al., 2020), play essential roles in regulating virulence gene expression and ensuring the pathogen’s ability to survive and proliferate within the host. Their coordinated action is vital for the bacterium’s pathogenicity and ability to evade host immune responses.

### Molecular insights into *Francisella*-autophagy interaction

The role of autophagy in the host defense against members of the *Francisella* genus is controversial (Qi et al., 2016). Comparative studies indicate that the less virulent LVS genome has undergone significant rearrangements compared to fully virulent SchuS4 strain. These rearrangements include inversions and deletions, leading to differences in gene content and organization. For instance, certain genes present in SchuS4 are either absent or pseudogenized in LVS, potentially affecting pathogenicity (Chaudhuri et al., 2007). While both strains possess the FPI, variations in gene sequences and expression levels have been observed, which may account for differences in their virulence (Faron et al., 2013). The distinct immune response dynamics observed between SchuS4 and LVS strains highlight differential regulation of key cellular processes, including the induction or suppression of autophagy, which may significantly impact their pathogenic strategies and host interactions. At an early stage of infection, *F. tularensis* dampens the autophagy process. The reasons why are still unanswered but a few possible explanations exist: a) *F. tularensis* prefers replication in cytosol instead of phagosome, b) the delay could bring time to become resistant to the autophagolysosome’s acidic environment. On the other hand, at late stages of infection, *F. tularensis* exploits autophagy to be hidden inside autophagosomes, which leads to suppression of proinflammatory cytokines production (Cremer et al., 2009). *F. tularensis* avoids autophagic degradation by escaping from the phagosome before it can be targeted by autophagic machinery. After being phagocytosed by host cells, *F. tularensis* rapidly escapes into the cytosol, thereby avoiding the lysosomal degradation pathway (Checroun et al., 2006). The bacterial factors that mediate this escape are crucial for avoiding recognition by the autophagy machinery. The study has shown that the IglC protein is essential for phagosomal escape and subsequent replication in the cytosol (Santic et al., 2005). PdpC and PdpD that were identified as T6SS effectors (Eshraghi et al., 2016) are required for phagosomal escape (Ludu et al., 2008; Uda et al., 2016) and OpiA, a phosphatidylinositol 3-kinase that is not encoded in FPI, is responsible for delaying phagosomal maturation (Ledvina et al., 2018). These effector proteins contribute to *Francisella* virulence (Brodmann et al., 2021). Similarly, many other studies have described various proteins that are necessary for the intracellular replication of *F. tularensis* inside the host cell (Bröms et al., 2010; Barel and Charbit, 2013; Celli and Zahrt, 2013; Ozanic et al., 2016; Alam et al., 2018; Spidlova et al., 2018; Stojkova et al., 2018). After *F. tularensis* enters the host cell (usually a macrophage), it is initially enclosed in a membrane-bound compartment known as the *Francisella*-containing vacuole. This is a phagosome-like structure formed during the phagocytosis process. Once *F. tularensis* escapes the FCV and replicates in the cytosol, the host cell may attempt to target the bacterium for destruction through autophagy, a process where cellular debris or pathogens are engulfed by autophagosomes (double-membrane structures) and delivered to lysosomes for degradation. *F. tularensis* interferes with the autophagy machinery by negative modulating the expression of autophagy-related genes and proteins, including BECN1, ATG5, ATG12, ATG16L2, ATG7, and ATG4a (Cremer et al., 2009). It is known that there exist two type of autophagy process: a) ATG5- dependent autophagy and b) ATG5-independent autophagy. In order to increase intracellular stocks of host amino acids, which may be utilized as a source of carbon, energy, and iron, *F. tularensis* induces ATG5-independent autophagy (Steele et al., 2013). It has been shown that WT strains of *Francisella* species are able to resist ATG5-dependent autophagy (Chong et al., 2012; Case et al., 2014). But it seems that the WT strains somehow exploit ATG5-dependent autophagy or responses to this pathway, as shown in an example of LVS. This strain proliferates less effectively in ATG5-deficient mice when compared to the WT mice (Kelava et al., 2020). Contrarily the mutant strains unable to replicate within the host or deficient in O-antigen synthesis are captured by ATG5-dependent autophagy (Case et al., 2014). Highly virulent strain *F. tularensis* subsp. *tularensis* SchuS4, which successfully avoids being recognized by the autophagic machinery, does not undergo ubiquitination (a critical step for autophagic targeting) in the cytosol and SchuS4 bacteria are not fully recognized by the key autophagy receptors p62/SQSTM1 and NBR1 (Chong et al., 2012), compared to the *F. tularensis* subsp. *holarctica* LVS that induces the recruitment of p62/SQSTM1 and LC3 already after 1 hour post infection (Härtlova et al., 2014). On the other hand, SchuS4 mutants that are not able to survive in the cytosol are tagged with ubiquitin and are subsequently captured into autophagosomes in a process dependent on ATG5, LC3, and p62/SQSTM1 (Chong et al., 2012). Tagging of bacteria by ubiquitin is a critical step for recognition in autophagic process and many bacteria can manipulate with this ubiquitination/deubiquitination system for their benefit (Vozandychova et al., 2021) and likewise *F. tularensis* that is able to suppress the activity of deubiquitinating enzymes and thus disrupt the homeostasis in ubiquitin cycle (Vozandychova et al., 2023). *F. tularensis* subsp. *holarctica* FSC200 downregulates the activity of USP10 enzyme in human macrophages 1 hour post infection, leading to the decreased amount/degradation of LC3, and thus repression of autophagy. USP10 normally removes ubiquitin molecule from the Beclin1 and LC3 (required for autophagosome formation) and thus these proteins are not degraded (because they are not tagged by ubiquitin). This suggests an active manipulation of the autophagy by *F. tularensis* specific strain (Vozandychova et al., 2023). *Francisella’s* HU protein (Stojkova et al., 2019), a DNA-binding protein involved in pathogenesis and virulence (Stojkova et al., 2018), may play a regulatory role in host´s response. It has already been shown in other pathogens that the bacterial HU protein is capable of binding host DNA (Stojkova and Spidlova, 2022). Since it has been demonstrated that *F. tularensis* HU protein is secreted into the medium (Konecna et al., 2010), we can speculate whether it enters the host cell, or even host nucleus where it could bind host DNA, because the *F. tularensis* HU protein´s DNA binding motif that we identified in our previous study (Pavlik and Spidlova, 2022) can be found in the host genome (Genome Data Viewer, NCBI). By binding to the promoter regions or regulatory elements of these genes, HU protein could affect their transcription, thus modulating the expression of key components in the autophagy process.

The key question remains regarding the dual role of autophagy as both a defense mechanism and a resource exploited by *Francisella* species. For instance, the exploitation of autophagy by *F. tularensis* at different infection stages, such as its ability to evade recognition by suppressing ubiquitination or manipulating ATG5-independent pathways, represents not just a mechanistic insight but a potential focal point for therapeutic intervention. So far, we know only a few of bacterial effector proteins that are somehow included in affecting the process of autophagy and their exact mechanisms of action remain elusive. It is crucial to delve deeper into the molecular interactions between bacterial effector proteins and host proteins that regulate autophagy machinery. Uncovering these detailed mechanisms can guide future research toward the development of targeted inhibitors, offering new strategies to combat infections.

## Conclusion

The study of *Francisella* provides general insight into the fight against intracellular bacterial pathogens, as many of these organisms share similar strategies for avoiding autophagy. Understanding the molecular interplay between host autophagy and microbial evasion tactics may be the basis for the development of treatments not only against tularemia but also against a number of other infectious diseases. Additionally, the insights gained from *F. tularensis* research have the potential to extend beyond infectious diseases and offer new approaches to manipulate autophagy for therapeutic benefit in cancer, autoimmune diseases, and other conditions where autophagy plays a critical role. Once the molecular interactions between *F. tularensis* and the host autophagy machinery are better understood, novel regulators of autophagy may be identified. These could include host proteins that are modulated by *F. tularensis* to suppress autophagy or new bacterial factors that inhibit autophagic processes. Future research should focus on uncovering the molecular details of *Francisella*-host interactions, characterizing the role of host genetic factors in autophagy response, and developing novel drugs and vaccine strategies that can modulate autophagy.

## References

[B1] Abd El Maksoud A. I. Elebeedy D. Abass N. H. Awad A. M. Nasr G. M. Roshdy T.. (2019). Methylomic changes of autophagy-related genes by legionella effector lpg2936 in infected macrophages. Front. Cell Dev. Biol. 7. doi: 10.3389/fcell.2019.00390 PMC6999459 32064256

[B2] Alam A. Golovliov I. Javed E. Sjöstedt A. (2018). ClpB mutants of *Francisella tularensis* subspecies holarctica and tularensis are defective for type VI secretion and intracellular replication. Sci. Rep. 8, 11324. doi: 10.1038/s41598-018-29745-4 30054549 PMC6063899

[B3] Arasaki K. Mikami Y. Shames S. R. Inoue H. Wakana Y. Tagaya M. (2017). Legionella effector Lpg1137 shuts down ER-mitochondria communication through cleavage of syntaxin 17. Nat. Commun. 8, 15406. doi: 10.1038/ncomms15406 28504273 PMC5440676

[B4] Ashida H. Mimuro H. Sasakawa C. (2015). Shigella manipulates host immune responses by delivering effector proteins with specific roles. Front. Immunol. 6. doi: 10.3389/fimmu.2015.00219 PMC4423471 25999954

[B5] Barel M. Charbit A. (2013). *Francisella tularensis* intracellular survival: To eat or to die. Microbes Infection. 989–997. doi: 10.1016/j.micinf.2013.09.009 24513705

[B6] Barnett T. C. Liebl D. Seymour L. M. Gillen C. M. Lim J. Y. LaRock C. N.. (2013). The globally disseminated M1T1 clone of Group A Streptococcus evades autophagy for intracellular replication. Cell Host Microbe 14, 675–682. doi: 10.1016/j.chom.2013.11.003 24331465 PMC3918495

[B7] Baxt L. A. Goldberg M. B. (2014). Host and bacterial proteins that repress recruitment of LC3 to shigella early during infection. PLoS One 9, e94653. doi: 10.1371/journal.pone.0094653 24722587 PMC3983221

[B8] Beck W. H. J. Kim D. Das J. Yu H. Smolka M. B. Mao Y. (2020). Glucosylation by the legionella effector setA promotes the nuclear localization of the transcription factor TFEB. iScience 23, 101300. doi: 10.1016/j.isci.2020.101300 32622269 PMC7334434

[B9] Benjamin J. L. Sumpter R. Levine B. Hooper L. V. (2013). Intestinal epithelial autophagy is essential for host defense against invasive bacteria. Cell Host Microbe 13, 723–734. doi: 10.1016/j.chom.2013.05.004 23768496 PMC3755484

[B10] Bergmann R. Gulotta G. Andreoni F. Sumitomo T. Kawabata S. Zinkernagel A. S.. (2022). The group A Streptococcus interleukin-8 protease SpyCEP promotes bacterial intracellular survival by evasion of autophagy. Infect. Microbes Dis. 4, 116–123. doi: 10.1097/im9.0000000000000098 37333426 PMC10275413

[B11] Bhogaraju S. Kalayil S. Liu Y. Bonn F. Colby T. Matic I.. (2016). Phosphoribosylation of ubiquitin promotes serine ubiquitination and impairs conventional ubiquitination. Cell 167, 1636–1649.e13. doi: 10.1016/j.cell.2016.11.019 27912065

[B12] Brodmann M. Schnider S. T. Basler M. (2021). Type VI secretion system and its effectors pdpC, pdpD, and opiA contribute to *Francisella* virulence in galleria mellonella larvae. Infection Immun. 89, e0057920. doi: 10.1128/iai.00579-20 PMC8208517 33875476

[B13] Bröms J. E. Sjöstedt A. Lavander M. (2010). The role of the *Francisella tularensis* pathogenicity island in type VI secretion, intracellular survival, and modulation of host cell signaling. Front. Microbiol. 1. doi: 10.3389/fmicb.2010.00136 PMC3109350 21687753

[B14] Case E. D. R. Chong A. Wehrly T. D. Hansen B. Child R. Hwang S.. (2014). The rancisella O-antigen mediates survival in the macrophage cytosol via autophagy avoidance. Cell. Microbiol. 16, 862–877. doi: 10.1111/cmi.12246 24286610 PMC4028363

[B15] Castillo E. F. Dekonenko A. Arko-Mensah J. Mandell M. A. Dupont N. Jiang S.. (2012). Autophagy protects against active tuberculosis by suppressing bacterial burden and inflammation. Proc. Natl. Acad. Sci. U. S. A. 109, E3168–E3176. doi: 10.1073/pnas.1210500109 23093667 PMC3503152

[B16] Celli J. (2019). The intracellular life cycle of *brucella* spp. Microbiol. Spectr. 7. doi: 10.1128/microbiolspec.bai-0006-2019 PMC6448592 30848234

[B17] Celli J. Zahrt T. C. (2013). Mechanisms of *Francisella tularensis* intracellular pathogenesis. Cold Spring Harbor Perspect. Med. 3, a010314. doi: 10.1101/cshperspect.a010314 PMC3683997 23545572

[B18] Chatterjee R. Chaudhuri D. Setty S. R. G. Chakravortty D. (2023). Deceiving the big eaters: Salmonella Typhimurium SopB subverts host cell xenophagy in macrophages via dual mechanisms. Microbes Infection 25, 105128. doi: 10.1016/j.micinf.2023.105128 37019426

[B19] Chaudhary A. Kamischke C. Leite M. Altura M. A. Kinman L. Kulasekara H.. (2018). [amp]]beta;-Barrel outer membrane proteins suppress mTORC2 activation and induce autophagic responses. Sci. Signaling 11, eaat7493. doi: 10.1126/scisignal.aat7493 30482849

[B20] Chaudhuri R. R. Ren C.-P. Desmond L. Vincent G. A. Silman N. J. Brehm J. K.. (2007). Genome sequencing shows that European isolates of *Francisella tularensis* subspecies tularensis are almost identical to US laboratory strain Schu S4. PLoS One 2, e352. doi: 10.1371/journal.pone.0000352 17406676 PMC1832225

[B21] Checroun C. Wehrly T. D. Fischer E. R. Hayes S. F. Celli J. (2006). Autophagy-mediated reentry of *Francisella tularensis* into the endocytic compartment after cytoplasmic replication. Proc. Natl. Acad. Sci. U. S. A. 103, 14578–14583. doi: 10.1073/pnas.0601838103 16983090 PMC1600002

[B22] Chong A. Wehrly T. D. Child R. Hansen B. Hwang S. Virgin H. W.. (2012). Cytosolic clearance of replication-deficient mutants reveals *Francisella tularensis* interactions with the autophagic pathway. Autophagy 8, 1342–1356. doi: 10.4161/auto.20808 22863802 PMC3442881

[B23] Choy A. Dancourt J. Mugo B. O’Connor T. J. Isberg R. R. Melia T. J.. (2012). The Legionella effector RavZ inhibits host autophagy through irreversible Atg8 deconjugation. Sci. (New York N.Y.) 338, 1072–1076. doi: 10.1126/science.1227026 PMC3682818 23112293

[B24] Claude-Taupin A. Bissa B. Jia J. Gu Y. Deretic V. (2018). Role of autophagy in IL-1β export and release from cells. Semin. Cell Dev. Biol. 83, 36–41. doi: 10.1016/j.semcdb.2018.03.012 29580970 PMC6173661

[B25] Clemens D. L. Ge P. Lee B.-Y. Horwitz M. A. Zhou Z. H. (2015). Atomic structure of T6SS reveals interlaced array essential to function. Cell 160, 940–951. doi: 10.1016/j.cell.2015.02.005 25723168 PMC4351867

[B26] Clemens D. L. Lee B.-Y. Horwitz M. A. (2004). Virulent and avirulent strains of *Francisella tularensis* prevent acidification and maturation of their phagosomes and escape into the cytoplasm in human macrophages. Infection Immun. 72, 3204–3217. doi: 10.1128/IAI.72.6.3204-3217.2004 PMC415696 15155622

[B27] Corkery D. P. Nadeem A. Aung K. M. Hassan A. Liu T. Cervantes-Rivera R.. (2021). Vibrio cholerae cytotoxin MakA induces noncanonical autophagy resulting in the spatial inhibition of canonical autophagy. J. Cell Sci. 134, jcs252015. doi: 10.1242/jcs.252015 33106317

[B28] Cremer T. J. Amer A. Tridandapani S. Butchar J. P. (2009). *Francisella tularensis* regulates autophagy-related host cell signaling pathways. Autophagy 5, 125–128. doi: 10.4161/auto.5.1.7305 19029814 PMC2774735

[B29] Dall’Armi C. Devereaux K. A. Di Paolo G. (2013). The role of lipids in the control of autophagy. Curr. biology: CB 23, R33–R45. doi: 10.1016/j.cub.2012.10.041 23305670 PMC3587843

[B30] Degabriel M. Valeva S. Boisset S. Henry T. (2023). Pathogenicity and virulence of *Francisella tularensis* . Virulence 14, 2274638. doi: 10.1080/21505594.2023.2274638 37941380 PMC10653695

[B31] Degtyar E. Zusman T. Ehrlich M. Segal G. (2009). A Legionella effector acquired from protozoa is involved in sphingolipids metabolism and is targeted to the host cell mitochondria. Cell. Microbiol. 11, 1219–1235. doi: 10.1111/j.1462-5822.2009.01328.x 19438520

[B32] De Leon J. A. Qiu J. Nicolai C. J. Counihan J. L. Barry K. C. Xu L.. (2017). Positive and Negative Regulation of the Master Metabolic Regulator mTORC1 by Two Families of Legionella pneumophila Effectors. Cell Rep. 21, 2031–2038. doi: 10.1016/j.celrep.2017.10.088 29166595 PMC5726772

[B33] Deretic V. Saitoh T. Akira S. (2013). ‘Autophagy in infection, inflammation and immunity’, Nature Reviews. Immunology 13, 722–737. doi: 10.1038/nri3532 24064518 PMC5340150

[B34] Dortet L. Mostowy S. Samba-Louaka A. Gouin E. Nahori M.-A. Wiemer E. A. C.. (2011). Recruitment of the major vault protein by InlK: a Listeria monocytogenes strategy to avoid autophagy. PLoS Pathog. 7, e1002168. doi: 10.1371/journal.ppat.1002168 21829365 PMC3150275

[B35] Dortet L. Mostowy S. Cossart P. (2012). Listeria and autophagy escape. Autophagy. 132–134. doi: 10.4161/auto.8.1.18218 22082958 PMC3335995

[B36] Eshraghi A.. (2016). Secreted effectors encoded within and outside of the *Francisella* pathogenicity island promote intramacrophage growth. Cell Host Microbe 20, 573–583. doi: 10.1016/j.chom.2016.10.008 27832588 PMC5384264

[B37] Faron M. Fletcher J. R. Rasmussen J. A. Long M. E. Allen L.-A. H. Jones B. D. (2013). The *Francisella tularensis* migR, trmE, and cphA genes contribute to F. tularensis pathogenicity island gene regulation and intracellular growth by modulation of the stress alarmone ppGpp. Infection Immun. 81, 2800–2811. doi: 10.1128/IAI.00073-13 PMC3719569 23716606

[B38] Feng Z.-Z. Jiang A.-J. Mao A.-W. Feng Y. Wang W. Li J.. (2018). The Salmonella effectors SseF and SseG inhibit Rab1A-mediated autophagy to facilitate intracellular bacterial survival and replication. J. Biol. Chem. 293, 9662–9673. doi: 10.1074/jbc.M117.811737 29610274 PMC6016468

[B39] Ganesan R. Hos N. J. Gutierrez S. Fischer J. Stepek J. M. Daglidu E.. (2017). *Salmonella Typhimurium* disrupts Sirt1/AMPK checkpoint control of mTOR to impair autophagy. PLoS Pathog. 13, e1006227. doi: 10.1371/journal.ppat.1006227 28192515 PMC5325604

[B40] Ge P. Lei Z. Yu Y. Lu Z. Qiang L. Chai Q.. (2022). *M. tuberculosis* PknG manipulates host autophagy flux to promote pathogen intracellular survival. Autophagy 18, 576–594. doi: 10.1080/15548627.2021.1938912 34092182 PMC9037497

[B41] Gradowski M. Pawłowski K. (2017). The Legionella pneumophila effector Lpg1137 is a homologue of mitochondrial SLC25 carrier proteins, not of known serine proteases. PeerJ 5, e3849. doi: 10.7717/peerj.3849 28966893 PMC5621508

[B42] Gutierrez M. G. Saka H. A. Chinen I. Zoppino F. C.M. Yoshimori T. Bocco J. L.. (2007). Protective role of autophagy against Vibrio cholerae cytolysin, a pore-forming toxin from V. cholerae. Proc. Natl. Acad. Sci. USA 104, 1829. doi: 10.1073/pnas.0601437104 17267617 PMC1794277

[B43] Hamasaki M. Furuta N. Matsuda A. Nezu A. Yamamoto A. Fujita N.. (2013). Autophagosomes form at ER-mitochondria contact sites. Nature 495, 389–393. doi: 10.1038/nature11910 23455425

[B44] Harris J. Hartman M. Roche C. Zeng S. G. O’Shea A. Sharp F. A.. (2011). Autophagy controls IL-1β Secretion by targeting pro-IL-1β for degradation. J. Biol. Chem. 286, 9587–9597. doi: 10.1074/jbc.M110.202911 21228274 PMC3058966

[B45] Härtlova A. Link M. Balounova J. Benesova M. Resch U. Straskova A.. (2014). Quantitative proteomics analysis of macrophage-derived lipid rafts reveals induction of autophagy pathway at the early time of *francisella tularensis* LVS infection. J. Proteome Res. 13, 796–804. doi: 10.1021/pr4008656 24364512

[B46] Hauser A. R. (2009). The type III secretion system of pseudomonas aeruginosa: infection by injection. Nature reviews. Microbiology 7, 654–665. doi: 10.1038/nrmicro2199 19680249 PMC2766515

[B47] Hernandez L. D. Pypaert M. Flavell R. A. Galán J. E. (2003). A Salmonella protein causes macrophage cell death by inducing autophagy. J. Cell Biol. 163, 1123–1131. doi: 10.1083/jcb.200309161 14662750 PMC2173598

[B48] Huang J. Brumell J. H. (2014). Bacteria–autophagy interplay: a battle for survival. Nat. Rev. Microbiol. 12, 101–114. doi: 10.1038/nrmicro3160 24384599 PMC7097477

[B49] Iula L. Keitelman I. A. Sabbione F. Fuentes F. Guzman M. Galletti J. G.. (2018). Autophagy mediates interleukin-1β Secretion in human neutrophils. Front. Immunol. 9. doi: 10.3389/fimmu.2018.00269 PMC5825906 29515581

[B50] Jia X. Knyazeva A. Zhang Y. Castro-Gonzalez S. Nakamura S. Carlson L.-A.. (2022). V. cholerae MakA is a cholesterol-binding pore-forming toxin that induces non-canonical autophagy. J. Cell Biol. 221, e202206040. doi: 10.1083/jcb.202206040 36194176 PMC9536202

[B51] Kaushik S. Cuervo A. M. (2018). The coming of age of chaperone-mediated autophagy. Nat. Rev. Mol. Cell Biol. 19, 365–381. doi: 10.1038/s41580-018-0001-6 29626215 PMC6399518

[B52] Kelava I. Mihelčić M. Ožanič M. Marečić V. Knežević M. Ćurlin M.. (2020). Atg5-deficient mice infected with *Francisella tularensis* LVS demonstrate increased survival and less severe pathology in internal organs. Microorganisms 8, 1531. doi: 10.3390/microorganisms8101531 33036147 PMC7600933

[B53] Khweek A. A. Caution K. Akhter A. Abdulrahman B. A. Tazi M. Hassan H.. (2013). A bacterial protein promotes the recognition of the Legionella pneumophila vacuole by autophagy. Eur. J. Immunol. 43, 1333–1344. doi: 10.1002/eji.201242835 23420491 PMC3782291

[B54] Kim J. Kundu M. Viollet B. Guan K.-L. (2011). AMPK and mTOR regulate autophagy through direct phosphorylation of Ulk1. Nat. Cell Biol. 13, 132–141. doi: 10.1038/ncb2152 21258367 PMC3987946

[B55] Kim J. K. Silwal P. Jo E.-K. (2020). Host-pathogen dialogues in autophagy, apoptosis, and necrosis during mycobacterial infection. Immune Network 20, e37. doi: 10.4110/in.2020.20.e37 33163245 PMC7609165

[B56] Klare I. Werner G. Witte W. (2001). Enterococci. Habitats, infections, virulence factors, resistances to antibiotics, transfer of resistance determinants. Contributions to Microbiol. 8, 108–122. doi: 10.1159/000060406 11764728

[B57] Konecna K. Hernychova L. Reichelova M. Lenco J. Klimentova J. Stulik J.. (2010). Comparative proteomic profiling of culture filtrate proteins of less and highly virulent *Francisella tularensis* strains. Proteomics 10, 4501–4511. doi: 10.1002/pmic.201000248 21136602

[B58] Kumar S. Gu Y. Abudu Y. P. Bruun J.-A. Jain A. Farzam F.. (2019). Phosphorylation of syntaxin 17 by TBK1 controls autophagy initiation. Dev. Cell 49, 130–144.e6. doi: 10.1016/j.devcel.2019.01.027 30827897 PMC6907693

[B59] Lauriano C. M. Barker J. R. Yoon S.-S. Nano F. E. Arulanandam B. P. Hassett D. J.. (2004). MglA regulates transcription of virulence factors necessary for *Francisella tularensis* intraamoebae and intramacrophage survival. Proc. Natl. Acad. Sci. U. S. A. 101, 4246–4249. doi: 10.1073/pnas.0307690101 15010524 PMC384726

[B60] Ledvina H. E. Kelly K. A. Eshraghi A. Plemel R. L. Peterson S. B. Lee B.. (2018). A phosphatidylinositol 3-kinase effector alters phagosomal maturation to promote intracellular growth of *Francisella* . Cell Host Microbe 24, 285–295.e8. doi: 10.1016/j.chom.2018.07.003 30057173 PMC6394229

[B61] Lemarignier M. Pizarro-Cerdá J. (2020). Autophagy and intracellular membrane trafficking subversion by pathogenic yersinia species. Biomolecules 10, 1637. doi: 10.3390/biom10121637 33291818 PMC7762052

[B62] Levine B. Kroemer G. (2019). Biological functions of autophagy genes: A disease perspective. Cell 176, 11–42. doi: 10.1016/j.cell.2018.09.048 30633901 PMC6347410

[B63] Li J. Qi L. Diao Z. Zhang M. Li B. Zhai Y.. (2022). Brucella btpB manipulates apoptosis and autophagic flux in RAW264.7 cells. Int. J. Mol. Sci. 23, 14439. doi: 10.3390/ijms232214439 36430916 PMC9693124

[B64] Ligeon L.-A. Moreau K. Barois N. Bongiovanni A. Lacorre D.-A. Werkmeister E.. (2014). Role of VAMP3 and VAMP7 in the commitment of Yersinia pseudotuberculosis to LC3-associated pathways involving single- or double-membrane vacuoles. Autophagy 10, 1588–1602. doi: 10.4161/auto.29411 25046114 PMC4206537

[B65] Lin D. Gao Y. Zhao L. Chen Y. An S. Peng Z. (2018). Enterococcus faecalis lipoteichoic acid regulates macrophages autophagy via PI3K/Akt/mTOR pathway. Biochem. Biophys. Res. Commun. 498, 1028–1036. doi: 10.1016/j.bbrc.2018.03.109 29551680

[B66] Louche A. Blanco A. Lacerda T. L.S. Cancade-Veyre L. Lionnet C. Bergé C.. (2023). Brucella effectors NyxA and NyxB target SENP3 to modulate the subcellular localisation of nucleolar proteins. Nat. Commun. 14, 102. doi: 10.1038/s41467-022-35763-8 36609656 PMC9823007

[B67] Ludu J. S. de Bruin O. M. Duplantis B. N. Schmerk C. L. Chou A. Y. Elkins K. L.. (2008). The *Francisella* pathogenicity island protein PdpD is required for full virulence and associates with homologues of the type VI secretion system. J. Bacteriology 190, 4584–4595. doi: 10.1128/JB.00198-08 PMC2446798 18469101

[B68] Mao K. Klionsky D. J. (2016). Xenophagy: A battlefield between host and microbe, and a possible avenue for cancer treatment. Autophagy 13, 223–224. doi: 10.1080/15548627.2016.1267075 PMC5324837 28026986

[B69] Maurin M. (2020). *Francisella tularensis*, tularemia and serological diagnosis. Front. Cell. Infection Microbiol. 10. doi: 10.3389/fcimb.2020.512090 PMC7649319 33194778

[B70] Mesquita F. S. Thomas M. Sachse M. Santos A. J. M. Figueira R. Holden D. W. (2012). The Salmonella deubiquitinase SseL inhibits selective autophagy of cytosolic aggregates. PLoS Pathog. 8, e1002743. doi: 10.1371/journal.ppat.1002743 22719249 PMC3375275

[B71] Mitchell G. Ge L. Huang Q. Chen C. Kianian S. Roberts M. F.. (2015). Avoidance of autophagy mediated by PlcA or ActA is required for Listeria monocytogenes growth in macrophages. Infection Immun. 83, 2175–2184. doi: 10.1128/IAI.00110-15 PMC4399067 25776746

[B72] Mitchell G. Cheng M. I. Chen C. Nguyen B. N. Whiteley A. T. Kianian S.. (2018). Listeria monocytogenes triggers noncanonical autophagy upon phagocytosis, but avoids subsequent growth-restricting xenophagy. Proc. Natl. Acad. Sci. USA 115, E210–E217. doi: 10.1073/pnas.1716055115 29279409 PMC5777066

[B73] Moreau K. Lacas-Gervais S. Fujita N. Sebbane F. Yoshimori T. Simonet M.. (2010). Autophagosomes can support Yersinia pseudotuberculosis replication in macrophages. Cell. Microbiol. 12, 1108–1123. doi: 10.1111/j.1462-5822.2010.01456.x 20180800

[B74] Nelson C. A. Murua C. Jones J. M. Mohler K. Zhang Y. Wiggins L.. (2019). *Francisella tularensis* transmission by solid organ transplantation 20171. Emerging Infect. Dis. 25, 767–775. doi: 10.3201/eid2504.181807 PMC6433034 30730826

[B75] Omotade T. O. Roy C. R. (2020). Legionella pneumophila excludes autophagy adaptors from the ubiquitin-labeled vacuole in which it resides. Infection Immun. 88, e00793–e00719. doi: 10.1128/IAI.00793-19 PMC7375760 32482642

[B76] Orenstein S. J. Cuervo A. M. (2010). Chaperone-mediated autophagy: Molecular mechanisms and physiological relevance. Semin. Cell Dev. Biol. 21, 719–726. doi: 10.1016/j.semcdb.2010.02.005 20176123 PMC2914824

[B77] Ozanic M. Marecic V. Lindgren M. Sjöstedt A. Santic M. (2016). Phenotypic characterization of the *Francisella tularensis* Δ*pdpC* and Δ*iglG* mutants. Microbes Infection 18, 768–776. doi: 10.1016/j.micinf.2016.07.006 27477000

[B78] Pavlik P. Spidlova P. (2022). Arginine 58 is indispensable for proper function of the *Francisella tularensis* subsp. holarctica FSC200 HU protein, and its substitution alters virulence and mediates immunity against wild-type strain. Virulence. 13, 1790–1809. doi: 10.1080/21505594.2022.2132729 36226562 PMC9578482

[B79] Pechous R. D. McCarthy T. R. Zahrt T. C. (2009). Working toward the future: insights into *Francisella tularensis* pathogenesis and vaccine development. Microbiol. Mol. Biol. reviews: MMBR 73, 684–711. doi: 10.1128/MMBR.00028-09 19946137 PMC2786580

[B80] Phalipon A. Sansonetti P. J. (2007). Shigella’s ways of manipulating the host intestinal innate and adaptive immune system: a tool box for survival? Immunol. Cell Biol. 85, 119–129. doi: 10.1038/sj.icb7100025 17213832

[B81] Pinotsis N. Waksman G. (2017). Crystal structure of the Legionella pneumophila Lpg2936 in complex with the cofactor S-adenosyl-L-methionine reveals novel insights into the mechanism of RsmE family methyltransferases. Protein Science: A Publ. Protein Soc. 26, 2381–2391. doi: 10.1002/pro.3305 PMC5699498 28940762

[B82] Price C. Jones S. Mihelcic M. Santic M. Abu Kwaik Y. (2020). Paradoxical pro-inflammatory responses by human macrophages to an amoebae host-adapted legionella effector. Cell Host Microbe 27, 571–584.e7. doi: 10.1016/j.chom.2020.03.003 32220647 PMC7224327

[B83] Pujol C. Klein K. A. Romanov G. A. Palmer L. E. Cirota C. Zhao Z.. (2009). Yersinia pestis can reside in autophagosomes and avoid xenophagy in murine macrophages by preventing vacuole acidification. Infection Immun. 77, 2251–2261. doi: 10.1128/IAI.00068-09 PMC2687347 19289509

[B84] Qi X. Man S. M. Malireddi R. K.S. Karki R. Lupfer C. Gurung P.. (2016). Cathepsin B modulates lysosomal biogenesis and host defense against *Francisella novicida* infection. J. Exp. Med. 213, 2081–2097. doi: 10.1084/jem.20151938 27551156 PMC5030800

[B85] Ramakrishnan G. (2017). Iron and virulence in *Francisella tularensis* . Front. Cell. Infection Microbiol. 7. doi: 10.3389/fcimb.2017.00107 PMC5378763 28421167

[B86] Rao L. De La Rosa I. Xu Y. Sha Y. Bhattacharya A. Holtzman M. J.. (2021). Pseudomonas aeruginosa survives in epithelia by ExoS-mediated inhibition of autophagy and mTOR. EMBO Rep. 22, e50613. doi: 10.15252/embr.202050613 33345425 PMC7857434

[B87] Rigard M. Bröms J. E. Mosnier A. Hologne M. Martin A. Lindgren L.. (2016). *Francisella tularensis* iglG belongs to a novel family of PAAR-like T6SS proteins and harbors a unique N-terminal extension required for virulence. PLoS Pathog. 12, e1005821. doi: 10.1371/journal.ppat.1005821 27602570 PMC5014421

[B88] Rolando M. Escoll P. Nora T. Botti J. Boitez V. Bedia C.. (2016). Legionella pneumophila S1P-lyase targets host sphingolipid metabolism and restrains autophagy. Proc. Natl. Acad. Sci. U. S. A. 113, 1901–1906. doi: 10.1073/pnas.1522067113 26831115 PMC4763766

[B89] Rowe H. M. Huntley J. F. (2015). From the outside-in: the *Francisella tularensis* envelope and virulence. Front. Cell. Infection Microbiol. 5. doi: 10.3389/fcimb.2015.00094 PMC4688374 26779445

[B90] Santic M. Molmeret M. Klose K. E. Jones S. Kwaik Y. A. (2005). The *Francisella tularensis* pathogenicity island protein IglC and its regulator MglA are essential for modulating phagosome biogenesis and subsequent bacterial escape into the cytoplasm. Cell. Microbiol. 7, 969–979. doi: 10.1111/j.1462-5822.2005.00526.x 15953029

[B91] Schmid D. Pypaert M. Münz C. (2007). Antigen-loading compartments for major histocompatibility complex class II molecules continuously receive input from autophagosomes. Immunity 26, 79–92. doi: 10.1016/j.immuni.2006.10.018 17182262 PMC1805710

[B92] Schnupf P. Portnoy D. A. (2007). isteriolysin O: a phagosome-specific lysin. Microbes Infection 9, 1176–1187. doi: 10.1016/j.micinf.2007.05.005 17720603

[B93] Seabaugh J. A. Anderson D. M. (2024). Pathogenicity and virulence of yersinia. Virulence 15, 2316439. doi: 10.1080/21505594.2024.2316439 38389313 PMC10896167

[B94] Shahnazari S. Namolovan A. Mogridge J. Kim P. K. Brumell J. H. (2011). Bacterial toxins can inhibit host cell autophagy through cAMP generation. Autophagy 7, 957–965. doi: 10.4161/auto.7.9.16435 21606683

[B95] Singh P. Subbian S. (2018). Harnessing the mTOR pathway for tuberculosis treatment. Front. Microbiol. 9. doi: 10.3389/fmicb.2018.00070 PMC5797605 29441052

[B96] Smith G. A. Marquis H. Jones S. Johnston N. C. Portnoy D. A. Goldfine H. (1995). The two distinct phospholipases C of Listeria monocytogenes have overlapping roles in escape from a vacuole and cell-to-cell spread. Infection Immun. 63, 4231–4237. doi: 10.1128/iai.63.11.4231-4237.1995 PMC173601 7591052

[B97] Spidlova P. Stojkova P. Dankova V. Senitkova I. Santic M. Pinkas D.. (2018). *Francisella tularensis* D-ala D-ala carboxypeptidase dacD is involved in intracellular replication and it is necessary for bacterial cell wall integrity. Front. Cell. Infection Microbiol. 8. doi: 10.3389/fcimb.2018.00111 PMC5903032 29692981

[B98] Spidlova P. Stojkova P. Sjöstedt A. Stulik J. (2020). Control of *Francisella tularensis* Virulence at Gene Level: Network of Transcription Factors. Microorganisms 8, 1622. doi: 10.3390/microorganisms8101622 33096715 PMC7588896

[B99] Spidlova P. Stulik J. (2017). *Francisella tularensis* type VI secretion system comes of age. Virulence 8, 628–631. doi: 10.1080/21505594.2016.1278336 28060573 PMC5626347

[B100] Starr T. Ng T. W. Wehrly T. D. Knodler L. A. Celli J. (2008). Brucella intracellular replication requires trafficking through the late endosomal/lysosomal compartment. Traffic (Copenhagen Denmark) 9, 678–694. doi: 10.1111/j.1600-0854.2008.00718.x 18266913

[B101] Steele S. Brunton J. Ziehr B. Taft-Benz S. Moorman N. Kawula T. (2013). *Francisella tularensis* Harvests Nutrients Derived via ATG5-Independent Autophagy to Support Intracellular Growth. PLoS Pathog. 9, e1003562. doi: 10.1371/journal.ppat.1003562 23966861 PMC3744417

[B102] Stojkova P. Spidlova P. Lenco J. Rehulkova H. Kratka L. Stulik J. (2018). HU protein is involved in intracellular growth and full virulence of *Francisella tularensis* . Virulence 9, 754–770. doi: 10.1080/21505594.2018.1441588 29473442 PMC5955460

[B103] Stojkova P. Spidlova P. (2022). Bacterial nucleoid-associated protein HU as an extracellular player in host-pathogen interaction. Front. Cell. Infection Microbiol. 12. doi: 10.3389/fcimb.2022.999737 PMC9445418 36081771

[B104] Stojkova P. Spidlova P. Stulik J. (2019). Nucleoid-associated protein HU: A lilliputian in gene regulation of bacterial virulence. Front. Cell. Infection Microbiol. 9. doi: 10.3389/fcimb.2019.00159 PMC6523023 31134164

[B105] Strong E. J. Ng T. W. Porcelli S. A. Lee S. (2021). Mycobacterium tuberculosis PE_PGRS20 and PE_PGRS47 proteins inhibit autophagy by interaction with rab1A. mSphere 6, e0054921. doi: 10.1128/msphere.00549-21. 34346699 PMC8386380

[B106] Tan T. Lee W. L. Alexander D. C. Grinstein S. Liu J. (2006). The ESAT-6/CFP-10 secretion system of Mycobacterium marinum modulates phagosome maturation. Cell. Microbiol. 8, 1417–1429. doi: 10.1111/j.1462-5822.2006.00721.x 16922861

[B107] Tattoli I. Sorbara M. T. Vuckovic D. Ling A. Soares F. Carneiro L. A. M.. (2012). Amino acid starvation induced by invasive bacterial pathogens triggers an innate host defense program. Cell Host Microbe 11, 563–575. doi: 10.1016/j.chom.2012.04.012 22704617

[B108] Thomas D. R. Newton P. Lau N. Newton H. J. (2020). Interfering with Autophagy: The opposing strategies deployed by legionella pneumophila and coxiella burnetii effector proteins. Front. Cell. Infection Microbiol. 10. doi: 10.3389/fcimb.2020.599762 PMC7676224 33251162

[B109] Torres A. Luke J. D. Kullas A. L. Kapilashrami K. Botbol Y. Koller A.. (2016). Asparagine deprivation mediated by *Salmonella asparaginase* causes suppression of activation-induced T cell metabolic reprogramming. J. Leukocyte Biol. 99, 387–398. doi: 10.1189/jlb.4A0615-252R 26497246 PMC4718193

[B110] Travis B. A. Ramsey K. M. Prezioso S. M. Tallo T. Wandzilak J. M. Hsu A.. (2021). Structural basis for virulence activation of *francisella tularensis* . Mol. Cell 81, 139–152.e10. doi: 10.1016/j.molcel.2020.10.035 33217319 PMC7959165

[B111] Uda A. Sharma N. Takimoto K. Deyu T. Koyama Y. Park E.. (2016). Pullulanase is necessary for the efficient intracellular growth of *Francisella tularensis* . PLoS One 11, e0159740. doi: 10.1371/journal.pone.0159740 27448164 PMC4957787

[B112] Valencia Lopez M. J. Schimmeck H. Gropengießer J. Middendorf L. Quitmann M. Schneider C.. (2019). Activation of the macroautophagy pathway by Yersinia enterocolitica promotes intracellular multiplication and egress of yersiniae from epithelial cells. Cell. Microbiol. 21, e13046. doi: 10.1111/cmi.13046 31099152

[B113] Van Kaer L. Parekh V. V. Postoak J. L. Wu L. (2019). Role of autophagy in MHC class I-restricted antigen presentation. Mol. Immunol. 113, 2–5. doi: 10.1016/j.molimm.2017.10.021 29126597 PMC5940586

[B114] Vozandychova V. Stojkova P. Hercik K. Rehulka P. Stulik J. (2021). The ubiquitination system within bacterial host–pathogen interactions. Microorganisms 9, 638. doi: 10.3390/microorganisms9030638 33808578 PMC8003559

[B115] Vozandychova V. Rehulka P. Hercik K. Spidlova P. Pavlik P. Hanus J.. (2023). Modified activities of macrophages’ deubiquitinating enzymes after *Francisella* infection. Front. Immunol. 14. doi: 10.3389/fimmu.2023.1252827 PMC10570801 37841261

[B116] Williams K. Gokulan K. Shelman D. Akiyama T. Khan A. Khare S. (2015). Cytotoxic mechanism of cytolethal distending toxin in nontyphoidal Salmonella serovar (Salmonella Javiana) during macrophage infection. DNA Cell Biol. 34, 113–124. doi: 10.1089/dna.2014.2602 25389664

[B117] Wong D. Bach H. Sun J. Hmama Z. Av-Gay Y. (2011). Mycobacterium tuberculosis protein tyrosine phosphatase (PtpA) excludes host vacuolar-H+–ATPase to inhibit phagosome acidification. Proc. Natl. Acad. Sci. U. S. A. 108, 19371–19376. doi: 10.1073/pnas.1109201108 22087003 PMC3228452

[B118] Wrench A. P. Gardner C. L. Siegel S. D. Pagliai F. A. Malekiha M. Gonzalez C. F.. (2013). MglA/sspA complex interactions are modulated by inorganic polyphosphate. PLoS One 8, e76428. doi: 10.1371/journal.pone.0076428 24116108 PMC3792966

[B119] Wu S. Shen Y. Zhang S. Xiao Y. Shi S. (2020). Salmonella interacts with autophagy to offense or defense. Front. Microbiol. 11. doi: 10.3389/fmicb.2020.00721 PMC7188831 32390979

[B120] Yamamoto H. Matsui T. (2024). Molecular mechanisms of macroautophagy, microautophagy, and chaperone-mediated autophagy. J. Nippon Med. School = Nippon Ika Daigaku Zasshi 91, 2–9. doi: 10.1272/jnms.JNMS.2024_91-102 37271546

[B121] Yoshikawa Y. Ogawa M. Hain T. Chakraborty T. Sasakawa C. (2009a). Listeria monocytogenes ActA is a key player in evading autophagic recognition. Autophagy 5, 1220–1221. doi: 10.4161/auto.5.8.10177 19855178

[B122] Yoshikawa Y. Ogawa M. Hain T. Yoshida M. Fukumatsu M. Kim M.. (2009b). Listeria monocytogenes ActA-mediated escape from autophagic recognition. Nat. Cell Biol. 11, 1233–1240. doi: 10.1038/ncb1967 19749745

[B123] Yuan K. Huang C. Fox J. Laturnus D. Carlson E. Zhang B.. (2012). Autophagy plays an essential role in the clearance of *Pseudomonas aeruginosa* by alveolar macrophages. J. Cell Sci. 125, 507–515. doi: 10.1242/jcs.094573 22302984 PMC3283879

[B124] Yuk J.-M. Yoshimori T. Jo E.-K. (2012). Autophagy and bacterial infectious diseases. Exp. Mol. Med. 44, 99–108. doi: 10.3858/emm.2012.44.2.032 22257885 PMC3296818

[B125] Zhang W. Dong C. Xiong S. (2024). Mycobacterial SapM hampers host autophagy initiation for intracellular bacillary survival via dephosphorylating Raptor. iScience 27, 109671. doi: 10.1016/j.isci.2024.109671 38646170 PMC11031826

[B126] Zheng Y. T. Shahnazari S. Brech A. Lamark T. Johansen T. Brumell J. H. (2009). The adaptor protein p62/SQSTM1 targets invading bacteria to the autophagy pathway. J. Immunol. (Baltimore Md.: 1950) 183, 5909–5916. doi: 10.4049/jimmunol.0900441 19812211

[B127] Zou J. Shankar N. (2014). Enterococcus faecalis infection activates phosphatidylinositol 3-kinase signaling to block apoptotic cell death in macrophages. Infection Immun. 82, 5132–5142. doi: 10.1128/iai.02426-14 PMC4249289 25267834

[B128] Zou J. Shankar N. (2016). The opportunistic pathogen resists phagosome acidification and autophagy to promote intracellular survival in macrophages. Cell. Microbiol. 18, 831–843. doi: 10.1111/cmi.12556 26663775

